# Disparity Indices and Overweight: Frame of Reference

**DOI:** 10.1089/heq.2019.0064

**Published:** 2019-12-13

**Authors:** Wendell C. Taylor, Casey P. Durand, Gregory Knell

**Affiliations:** Department of Health Promotion and Behavioral Sciences, Center for Health Promotion and Prevention Research, School of Public Health, The University of Texas Health Science Center at Houston, Houston, Texas.

**Keywords:** disparity, body mass index, income, obesity

## Abstract

**Purpose:** The purpose of this study was to compare state rankings of body mass index (BMI) among three different indices of income disparities (i.e., low-, middle-, and high-income thresholds) and BMI. One measure of disparities was based on national standards and the other measure was based on state-specific data.

**Methods:** Data were from the 2016 Behavioral Risk Factor Surveillance System and analyzed in 2018. To assess differences between the two indices, Spearman Rank Order Correlation coefficient with a Bonferroni adjustment and kappa statistic were used.

**Results:** Spearman rank-order correlation coefficient with a Bonferroni adjustment found that the two indices had a very weak monotonic relationship (ρ=0.11, *p*=0.46). The kappa value [κ (df=48)=0.02, *p*-value=0.43] revealed the indices were not concordant. The rankings of states based on national and state-specific disparity indices were distinctly different.

**Conclusion:** Our study highlights the importance of choosing disparity indices. To analyze state similarities and differences, findings and interpretations are different when using a national standard applied to all states versus state-specific data as the frame of reference for the disparity index. Future research is needed to confirm the generalizability of our findings. In addition to income, our approach can be used with other sociodemographic variables such as age, race/ethnicity, sex, and education. The overall goal is to present a comprehensive and nuanced perspective of disparities contributing to the overweight/obesity epidemic.

## Introduction

One measure of overweight and obesity is thresholds or categories of body mass index (BMI) calculated as weight in kilograms divided by height in meters squared, rounded to one decimal place. For most studies, overweight is defined as 25.0–29.9 and obesity in adults is defined as BMI of ≥30.^1^ In 2015–2016, the prevalence of overweight/obesity among U.S. adults was 39.8%^1^; another study reported that 5% of U.S. citizens are morbidly obese.^2^ Overweight/obesity is a major contributor to type 2 diabetes, hypertension, and coronary heart disease, as well as other chronic conditions.^3^

Health disparities are health differences associated with social, economic, and/or environmental disadvantage and adversely affect people who typically experience greater obstacles to achieving and sustaining optimal health.^4^ There are demographic and socioeconomic disparities (e.g., race and/or ethnicity, education, income, gender, and geographic location) that characterize the overweight/obesity epidemic.^5^ An Institute of Medicine's objective is to eliminate disparities related to weight status, which include documenting existing disparities and monitoring progress to reduce disparities.^6^ Disparity indices are essential for accurate data monitoring and reporting.

There are no known studies that directly compared different frames of reference for disparity indices.^7–14^ Given that disparity is a multidimensional construct,^7^ to compare different frames of reference is useful and can provide insights into which measures are appropriate for a research study. Frames of reference can be a national standard or state-specific standard, which means data derived from the place of interest. The frames of reference can lead to different interpretations. The concordance and discordance between different frames is important information.

In a study that applied the Healthy Weight Disparity Index (HWDI), body mass indices and income disparities measures (differences among BMI among low-, middle-, and high-income thresholds) were used.^12^ The 50 states and Washington District of Columbia (D.C.) were ranked from lowest to highest disparities based on these variables.^12^ The specific algorithm used to calculate this index was as follows: HWDI (for income)=(24.9 − high income BMI) + (middle income BMI −24.9) + (low income BMI −24.9).^12^ BMI of 24.9 was chosen because according to the Centers for Disease Control and Prevention (CDCP), BMI from >18.5 to ≤24.9 is a healthy weight, >25.0 to ≤29.9 is overweight, and ≥30 is obese. Based on the literature, the high-income group would most likely have the healthiest BMI (therefore, we use the high-income group as the reference group).^15^

The standard BMI >18.5 to ≤24.9 was applied to all states and Washington D.C. Similarly, the same income thresholds were applied to all states and Washington D.C. Total household income was low (<$35,000 per year), middle ($35,000 to <$75,000), and high ($75,000 or more).^12,16^ Overweight/obesity disparities relate to gender, race and/or ethnicity, education, income, and geographic location.^5^ The HWDI can be adapted to other overweight-/obesity-related disparities (e.g., race and/or ethnicity, education, income, and gender). As noted earlier, income was chosen for the original HWDI because empirical studies document that income is a primary disparity related to overweight/obesity.^15,17^

The original HWDI algorithm applied the same standards for income and weight status to all states and Washington D.C. State-specific data as the frame of reference demonstrate flexibility and utility of the HWDI algorithm. It can be argued that individualizing the income levels and BMI values based on state-specific data can provide more meaningful comparisons among states. The rationale for this argument is that states differ in BMI and income. For example, the standard thresholds of income: low (<$35,000 per year), middle ($35,000 to <$75,000), and high ($75,000 or more) may not accurately reflect the low-, middle-, and high-income thresholds of each state. Therefore, individualizing income thresholds and BMI for each state enables comparisons that are perhaps more relevant and tailored to each location.

The original HWDI can be revised by using the mean BMI of each state as the reference for that state and similarly, income can be individualized per state. Therefore, the rankings of states by the revised HWDI (RHWDI) is based on state-specific data instead of standard thresholds for all states and Washington D.C. To assess the viability and usefulness of the RHWDI, the objectives of this study were as follows:

1. To develop an RHWDI based on state-specific data, income, and BMI;
2. To rank states from lowest to highest based on RHWDI;
3. To compare state rankings between RHWDI and the original HWDI.

Testing feasibility, versatility, and robustness of disparity indices related to weight status advances the field and ultimately can assist in developing and monitoring effective interventions to address the overweight/obesity crisis. Tracking the trajectory of income disparities in overweight/obesity is important to policy makers as well as to evaluate population-level and policy interventions designed to eliminate disparities.^6^

## Methods

### Data source

Data were from the 2016 Behavioral Risk Factor Surveillance System (BRFSS). The BRFSS is an ongoing, annual phone-based survey of adults (18 and over) in the United States conducted by the CDCP, designed to capture information on a broad range of health-related topics. The BRFSS samples households with landlines as well as cell phone-only households, and uses a complex sampling design to produce estimates that are representative of the noninstitutionalized population of U.S. adults. Further details can be found elsewhere.^18^ BRFSS respondents were excluded if they met at least one of the following criteria: pregnant; older than 65 years; or BMI <18.5 (underweight). These criteria were applied because it has been found that body composition stabilizes between 20 and 60–70 years of age,^18,19^ and those with a BMI of <18.5 kg/m^2^ may be suffering from an underlying medical condition.^20^ For this research, Institutional Review Board approval was not required because there was no contact with human subjects.

### Variables

The following variables were included in the analysis: age (ordinal, coded in five categories: 18–24, 25–34, 35–44, 45–54, and 55–64); household income (ordinal, coded in eight categories: <$10,000, $10,000 to <$15,000, $15,000 to <$20,000, $20,000 to <$25,000, $25,000 to <$35,000, $35,000 to <$50,000, $50,000 to <$75,000, and ≥$75,000); sex (binary, coded in two categories: male or female); race/ethnicity (nominal, coded in six categories: white only, non-Hispanic; black only, non-Hispanic; Asian only, non-Hispanic; other race only, non-Hispanic; multiracial, non-Hispanic, and Hispanic); education (ordinal, coded in four categories): less than high school graduate, high school graduate, some college or technical school, or college graduate; number of adults in the household and number of children in the household (both continuous); and BMI (continuous, computed by Centers for Disease Control and Prevention based on self-reported height and weight).

There are no universally accepted definitions of low-, middle-, and high-income thresholds. Therefore, we chose the most conservative approach based on the ordinal categories presented in the BRFSS data and the best available evidence. Consistent with previous literature, we believe that the middle income (185% of poverty guidelines) based on the eligibility of state and federal assistance programs, and 75th percentile as the high-income category in our study is justified.^21^ In addition, our income categories provide similar sample sizes for analyses, which align with Ogden's approach.

For this analysis, we first calculated the ratio of the participant's reported household income to the poverty guidelines as established by the United States Department of Health and Human Services for a household of that size as of January 2016. We defined low income as those households whose household income and size put them at or below 185% of the poverty guidelines. We chose this approach as the demarcation between low- and middle-income thresholds because individuals from households at or below this level are eligible for a number of state and federal assistance programs, including Women, Infants, and Children, Medicaid, Children's Health Insurance Program, and Head Start. Middle-income threshold was defined as individuals from households making >185% of the poverty guidelines, but less than or equal to the state-specific 75th percentile of the distribution of the household income to poverty-level ratio. High-income threshold was defined as individuals from households whose poverty guideline ratio was greater than the state-specific 75th percentile. The BRFSS reports income as a categorical variable with ordinal values that each correspond to a specific income range. To calculate the income poverty guideline ratio, we assigned each person an income that reflected the highest value possible in that range (e.g., those in the <$10,000 category were assigned an income of $9999); those in the highest income category (≥$75,000) were assigned an income of $75,000. This method was chosen because it presents the most optimistic scenario for household income; it places more individuals in the middle- and high-income categories than if a different method were used (e.g., assigning the lowest possible income for a category, or the middle value). As such, this method represents the most conservative approach; the RHWDI for each state would likely be larger (i.e., greater disparities) if a different method were used.

### Statistical analysis

The dataset contained nonignorable amounts of missing data. To address this challenge, multiple imputations by chained equations with 20 imputed datasets were used. Continuous variables (BMI, children in household, and adults in household) were imputed using linear regression, ordinal variables (education and income) were imputed using predictive mean matching, and dichotomous variables (sex) were imputed using logistic regression. Due to difficulties in achieving imputation convergence, nominal variables (race/ethnicity) were not imputed; observations missing on any of these variables were subject to case-wise deletion. Age was not missing for any observation because it was previously imputed by CDCP if a respondent did not report their age. Imputations were conducted separately for each state. Finally, the sampling weight was included as a predictor of missingness in the imputation models. Imputed datasets were used in all subsequent analyses.

To calculate state-specific adjusted BMI means, we regressed BMI on age, race/ethnicity, sex, and education, using that model to compute *post-hoc* adjusted means. The same model was used to compute adjusted mean BMI within each of the three income categories for each state. All models appropriately accounted for the complex sampling nature of the BRFSS. Analyses were conducted in 2018 using Stata 15.1 (StataCorp LLC, College Station, TX).

Using the adjusted means as inputs, the formula to calculate the state-specific RHWDI was as follows: (State-average BMI − high-income BMI) + (middle-income BMI − state-average BMI) + (low-income BMI − state-average BMI). This revised formula is different from the original formula (HWDI) because it assesses the effects of state averages of income and weight status in contrast to national income and standard BMI thresholds reflected in the original formula.

### Comparison of indices

To assess differences between the two indices, Spearman rank-order correlation coefficient with a Bonferroni adjustment and kappa statistic were used.

## Results

### Demographics

Participants were primarily non-Hispanic whites (59%) and females (51%); 59% had at least some college education and 34.2% had an annual household income of at least $75,000 (Table 1). The mean (standard deviation) BMI of the study participants was 28.2 (0.02).

**Table 1.** Sociodemographic Characteristics of Participants from the 2016 Behavioral Risk Factor Surveillance System (*N*=196,073,744)

Variable	%
Age, years	
18–24	15.2
25–34	21.3
35–44	20.6
45–54	21.6
55–64	21.3
Race/ethnicity	
White, non-Hispanic	58.5
Black, non-Hispanic	12.2
Asian, non-Hispanic	5.5
Other race, non-Hispanic	1.6
Multiracial, non-Hispanic	1.6
Hispanic	18.6
Sex	
Male	49.2
Female	50.8
Education	
Less than high school	13.4
HS graduate	27.7
Some college or technical school	31.5
College graduate	27.3
Annual household income	
<$10,000	6.2
$10,000 to <$15,000	5.1
$15,000 to <$20,000	7.6
$20,000 to <$25,000	8.9
$25,000 to <$35,000	9.8
$35,000 to <$50,000	12.9
$50,000 to <$75,000	14.7
≥$75,000	34.2
Body mass index, mean (SE)	28.2 (0.02)
% of the poverty level, mean (SE)	586.8 (20.7)

HS, high school; SE, standard error.

### RHWDI by state

RHWDI values ranged from 0.004 to 2.11 (Table 2). States with the lowest RHWDI values were Nevada (0.004), South Carolina (0.01), Ohio and North Carolina (0.06), and New Hampshire (0.12). States with the greatest RHWDI values were Rhode Island (2.11), Arkansas (2.08), Oregon (1.95), West Virginia (1.84), and Maryland (1.79). Twenty-one states and the D.C., including some of those at the extreme ends (i.e., Nevada, Ohio, and Arkansas), had negative RHWDI values, which indicate a greater mean BMI value among high-income groups, compared to middle- and low-income groups. Due to data concerns, RHWDI values were not computed for Michigan (>50% missing for number of adults in household variable) and South Dakota (presence of zero cells in the income category/race crosstab).

**Table 2.** Revised Healthy Weight Disparity Index Values and Rankings by State, Behavioral Risk Factor Surveillance System, 2015

State	RHWDI	RHWDI (rank)	HWDI	HWDI (rank)
Nevada	0.00^a^	1	1.03	1
South Carolina	0.01	2	3.81	45
Ohio	0.06^a^	3	2.95	24
North Carolina	0.06	4	3.75	41
New Hampshire	0.12	5	2.70	18
Maine	0.14	6	2.75	20
Indiana	0.18^a^	7	3.77	42
Delaware	0.23^a^	8	3.29	31
Kansas	0.24	9	4.02	46
Alabama	0.25^a^	10	3.02	27
Utah	0.26	11	1.84	4
Colorado	0.27^a^	12	2.28	10
Louisiana	0.33^a^	13	3.59	37
Iowa	0.33	14	3.29	32
Oklahoma	0.33	15	3.45	34
Alaska	0.37^a^	16	2.54	16
Florida	0.38	17	2.75	21
Arizona	0.42^a^	18	2.11	9
Montana	0.43^a^	19	2.10	7
District of Columbia	0.48^a^	20	4.08	48
Minnesota	0.52^a^	21	2.40	13
Idaho	0.56^a^	22	2.77	22
New York	0.66	23	2.74	19
Tennessee	0.71^a^	24	2.35	12
California	0.74	25	2.10	8
Georgia	0.74^a^	26	3.77	43
New Mexico	0.77^a^	27	1.67	2
Wyoming	0.77^a^	28	3.07	28
Massachusetts	0.78	29	2.40	14
Washington	0.79	30	3.11	29
Virginia	0.88	31	3.66	38
North Dakota	0.91	32	2.45	15
Texas	0.92	33	3.77	44
New Jersey	0.94	34	2.03	6
Pennsylvania	0.97	35	3.46	35
Mississippi	1.08^a^	36	4.35	49
Connecticut	1.09^a^	37	2.31	11
Missouri	1.18^a^	38	3.72	40
Illinois	1.20^a^	39	4.03	47
Vermont	1.22	40	3.01	26
Nebraska	1.27	41	3.70	39
Hawaii	1.47	42	1.73	3
Wisconsin	1.49	43	3.00	25
Kentucky	1.62	44	2.88	23
Maryland	1.79	45	3.42	33
West Virginia	1.84	46	2.02	5
Oregon	1.95	47	3.51	36
Arkansas	2.08^a^	48	3.26	30
Rhode Island	2.11	49	2.67	17

Michigan and S. Dakota were not included in the analysis due to a large amount of missing data in the variables used to calculate household income.

^a^ Denotes a negative RHDWI value, which indicates a greater mean BMI value among high-income groups, compared to middle- and low- income groups.

BMI, body mass index; RHWDI, revised healthy weight disparity index; HWDI, healthy weight disparity index.

### Comparison of HWDI and RHWDI

The RHWDI rankings were compared to the previously published HWDI using the Spearman rank-order correlation coefficient with a Bonferroni adjustment, and it was found that the indices had a very weak monotonic relationship (ρ=0.01, *p*=0.45). To further evaluate the comparability of the two indices, RHWDI and the original HWDI were placed into terciles and compared using a kappa statistic. The kappa value [κ (df=48)=0.09, *p*-value=0.34] revealed the indices were not concordant. Five states (Nevada, Alaska, Wyoming, Idaho, and Pennsylvania) were ranked the same for both indices, Washington differed by one, and three states (Colorado, Missouri, and Nebraska) differed by two ranking positions (Table 3). Furthermore, 38 states had a difference in ranking position of 5 or more indicating >10% change. The greatest differences in rankings were observed for South Carolina (43 rankings), West Virginia (41 rankings), Hawaii (39 rankings), and Kansas and North Carolina (37 rankings).

**Table 3.** Comparison of Revised Healthy Weight Disparity Index and Health Weight Disparity Index by State, Behavioral Risk Factor Surveillance System, 2015

Light gray shaded—low rank; gray shaded—middle rank; dark gray shaded—high rank.

^a^ RHWDI rank is the numerical order for each state by RHWDI; HWDI rank is the numerical order for each state by the previous HWDI; low, middle, and high reflect tercile categories based on ranks for all states, excluding Michigan and South Dakota, which were not included in the analysis due to a large amount of missing data in the variables used to calculate household income; difference is the difference in rank order between the RHWDI and HWDI indices.

Diff, difference.

## Discussion

### Summary of results

The objectives of this research were to develop an RHWDI, rank 50 states and Washington D.C., and compare state rankings of the original HWDI to the RHWDI. The research objectives were achieved and we found that the rankings were not concordant. This absence of agreement indicates that indices derived from state-specific reference points (i.e., within the geographic area for cross-site comparisons) will provide dramatically different results than indices derived from a uniform or standard reference point. To further underscore the differences between the two indices, the RHWDI had 19 negative values, which means that high-income individuals had greater BMI values than low-income individuals. In contrast, the original HWDI had no negative values.

In future research, careful deliberations and a strong rationale should be the basis for deciding whether a standard or state-specific reference frame is more appropriate. If a national standard is used, then the disparity is relative to the nation. Reference points derived from within the city or geographic unit of analyses enable cross-site comparisons. Furthermore, to retain consistency, the same type of index should be used to document trends over time, ascertaining increasing or diminishing disparities.

### Comparison to previous literature

For a given geographic area, the Index of Concentration at the Extremes (ICE) simultaneously measures concentrations of privilege and privation. ICE was used to investigate risk of hypertension in a predominantly working-class study population.^22^ ICE measures (at the census track level) assess extreme concentrations of both income and racial/ethnic composition. Income thresholds were defined as low (<US$20000 annual household income) and high income (>US$100 000) based on 2010 United States Census data and the 20th and 80th centiles of the national household income distribution.^22^ In contrast, in our study, income categories accounted for state-specific variations instead of one uniform standard.

In another study, based on self-report, the poverty to income ratio was used as the income measure.^13^ This approach accounts for inflation and household composition, but does not account for regional variation in prices. In our study, we accounted for state-specific variation in income and BMI.

### Study limitations and strengths

Data were used from the United States 2016 BRFSS, which is a probability sample of the U.S. noninstitutionalized population. Military personnel on active duty, patients in mental facilities, and older adults who are institutionalized were excluded. Survey participants' height and weight were self-reported; therefore, there are concerns related to measurement error and social desirability bias.^23^ A primary concern is reporting bias in height and/or weight (used to calculate BMI) across population subgroups such as gender, race and/or ethnicity, body weight status, income, age, and geography.^24,25^ If substantial self-report biases related to sociodemographic variables are confirmed, then more objective assessments should be used. Even with these limitations, BRFSS is the only known data set with the relevant national data for our study.

Another concern is missing data. If nonresponders had greater overweight/obesity prevalence and lower income levels, the order of the rankings could be different. In addition, in our observational and cross-sectional study, the association between income and overweight should be not interpreted as causal.

This study has several strengths. It is the only known study that specifically compared the rankings of states by income and overweight by two different approaches—state-specific versus national reference frames. The original HWDI and the RHWDI provide a unique opportunity to test two different approaches. Several analytic approaches were used to impute missing data. Different analytic measures were used to compare and contrast the two rankings to provide confirmatory evidence of the findings. Furthermore, age, race/ethnicity, sex, and education were controlled for. The only known national data set with relevant data was used in our study.

## Conclusions

We used the eco-social theory of disease distribution as a guiding framework for understanding health inequities^22^; this theory emphasizes the social and ecological context of individuals and how these contexts relate to health outcomes. Based on the availability of data, smaller geographic areas such as counties, cities, census tracts, census block groups, municipal planning districts, or ZIP codes can be considered to better embody the eco-social theory of disease distribution. Furthermore, to get a full picture of the complexity of overweight/obesity trends, researchers must examine socioeconomic disparities over time within racial and ethnic groups, as well as gender.^13,22^

With some exceptions, long-term trends (1960–2008) have documented that increases in overweight/obesity, severe obesity, and BMI are similar across different racial/ethnic, educational, and income groups. In other words, increases in excess weight were not limited to certain subgroups. These authors concluded, “obesity in the United States is a true epidemic” affecting the entire society.^13^ Another study, examining adult BMI, obesity, and morbid obesity trends from 1971 to 2012, concluded that individual increases in weight status were widely distributed across all age groups and birth cohorts.^14^ Furthermore, during the past three decades, there has been an increase in overweight/obesity rates among all education subgroups.^26^

Our study highlights the importance of choosing disparity indices. To analyze state similarities and differences, findings and interpretations are different when using a national standard applied to all states versus state-specific data as the frame of reference for the disparity index. From an intervention perspective, the urgent public health challenge is to embrace successful and sustainable societal-level interventions to promote healthy lifestyles that will reverse the upward overweight/obesity trend in all population subgroups. The population-based approach and the vulnerable population approach can be complementary.^27^

## Abbreviations Used

BMI: body mass index
BRFSS: Behavioral Risk Factor Surveillance System
CDCP: Centers for Disease Control and Prevention
D.C.: District of Columbia
HWDI: Healthy Weight Disparity Index
ICE: Index of Concentration at the Extremes
RHWDI: revised HWDI
